# Understanding the Thermal Properties of Precursor-Ionomers to Optimize Fabrication Processes for Ionic Polymer-Metal Composites (IPMCs)

**DOI:** 10.3390/ma11050665

**Published:** 2018-04-25

**Authors:** Sarah Trabia, Kisuk Choi, Zakai Olsen, Taeseon Hwang, Jae-Do Nam, Kwang J. Kim

**Affiliations:** 1 Active Materials and Smart Living (AMSL) Laboratory, Mechanical Engineering Department, University of Nevada—Las Vegas, Las Vegas, NV 89154-4027, USA; sarah.trabia@unlv.edu (S.T.); olsenz@unlv.nevada.edu (Z.O.); taeseon.hwang@unlv.edu (T.H.); 2 Department of Polymer Science and Engineering, Division of Applied Chemistry, Sungkyunkwan University, Suwon 440-746, Korea; kisuk929@skku.edu (K.C.); jdnam@skku.edu (J.-D.N.)

**Keywords:** precursor ionomers, material characteristics, Nafion, Aquivion, 3D printing

## Abstract

Ionic polymer-metal composites (IPMCs) are one of many smart materials and have ionomer bases with a noble metal plated on the surface. The ionomer is usually Nafion, but recently Aquivion has been shown to be a promising alternative. Ionomers are available in the form of precursor pellets. This is an un-activated form that is able to melt, unlike the activated form. However, there is little study on the thermal characteristics of these precursor ionomers. This lack of knowledge causes issues when trying to fabricate ionomer shapes using methods such as extrusion, hot-pressing, and more recently, injection molding and 3D printing. To understand the two precursor-ionomers, a set of tests were conducted to measure the thermal degradation temperature, viscosity, melting temperature, and glass transition. The results have shown that the precursor Aquivion has a higher melting temperature (240 °C) than precursor Nafion (200 °C) and a larger glass transition range (32–65 °C compared with 21–45 °C). The two have the same thermal degradation temperature (~400 °C). Precursor Aquivion is more viscous than precursor Nafion as temperature increases. Based on the results gathered, it seems that the precursor Aquivion is more stable as temperature increases, facilitating the manufacturing processes. This paper presents the data collected to assist researchers in thermal-based fabrication processes.

## 1. Introduction

Ionic polymer-metal composites (IPMCs) are one of the smart materials that have been studied by many researchers [1,2,3,4]. IPMCs have an ionomer base with a noble metal plated onto the surface. When a voltage is applied to the IPMC, the cations within the membrane are pulled towards the negatively charged side, causing it to swell and forcing a bending motion. The smart material can also act as a sensor when it is deformed [5,6,7]. The movement of the cations within an ionomer can generate a small amount of voltage that can be amplified.

**Figure 1:**
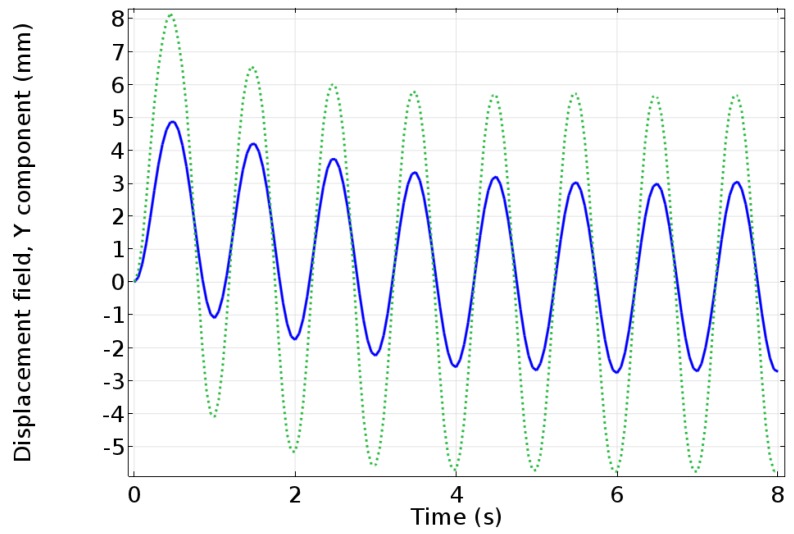
COMSOL Multi-physics simulation of a parameter sweep to predict the actuation capabilities of a Nafion-based IPMC (solid line) and an Aquivion-based IPMC (dotted line). The applied voltage is 0.5 V at a frequency of 1 Hz. The IPMC modeled is 51.07 mm long, 9.94 mm wide, and 0.586 mm thick. The model has two components: the transport, electric current, and the general form of partial differential equation (PDE) were used to model Poisson-Nernst-Planck system, which was coupled to a model for the solid mechanics to obtain the tip deflection [20].

**Figure 2:**
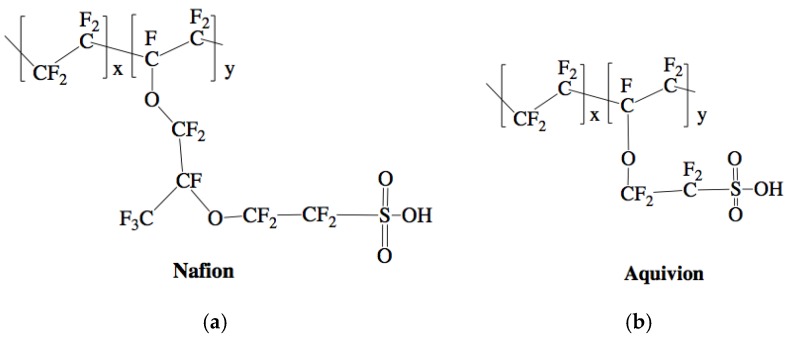
Chemical structure of Nafion (**a**) and Aquivion (**b**) activated, membrane forms [21,22].

**Figure 3:**
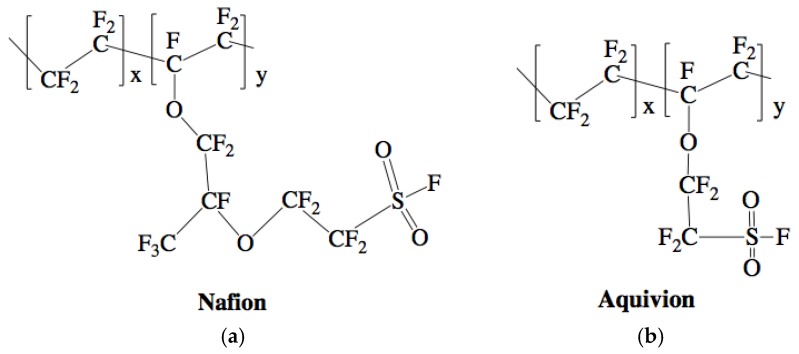
Chemical structure of Nafion (**a**) and Aquivion (**b**) un-activated, precursor forms [23,24].

Nafion is usually the ionomer chosen for IPMCs. Nafion is an ideal option, as it is chemically stable and has good conductivity and material properties [8,9,10]. Many have studied ways to improve the properties of Nafion. However, the most critical characteristic of Nafion is its ionic conductivity and ion exchange capacity. Since there has not been a way to change this value directly other than in-depth chemistry approaches, a convenient solution is to choose another ionomer with a higher ionic conductivity and ionic exchange capacity. Recently, Aquivion has been shown to increase the IPMC’s performance overall because it has a higher ionic conductivity and ionic exchange capacity [11]. The resulting performance measure is also estimated by a physic-based modeling (Figure 1) where it can be seen that as the diffusion coefficient increases, due mainly to the increase in ion conductivity, the actuation abilities of IPMCs increase. Both ionomers are available in two forms: activated and un-activated. The activated forms of the ionomers have a tetrafluoroethylene backbone, with Nafion having a perfluorovinyl ether side group and a sulfonate end group [12] and Aquivion having a double ether perfluoro side chain and sulfonic acid end group [13] (Figure 2). The acid end group is what deems the ionomers to be “activated” and able to conduct ions, but also means the ionomers are unable to melt [14,15]. Their thermal properties have been studied thoroughly by researchers such as de Almeida and Kawano as well as Zhao and Benziger [16,17]. The un-activated (precursor) forms of the ionomers have the following structure: tetrafluoroethylene backbone, with precursor Nafion having a perfluoro-3,6-dioxa-4-methyl-7-octenesulfonyl fluoride side chain, while precursor Aquivion has a shorter sulfonyl fluoride vinyl ether side chain (Figure 3). This different end group allows the precursor ionomer to melt, but cannot conduct ions [15,18]. It is imperative to hydrolyze the ionomers to switch the end group to an acid form once the shape is made [19]. 

**Figure 4:**
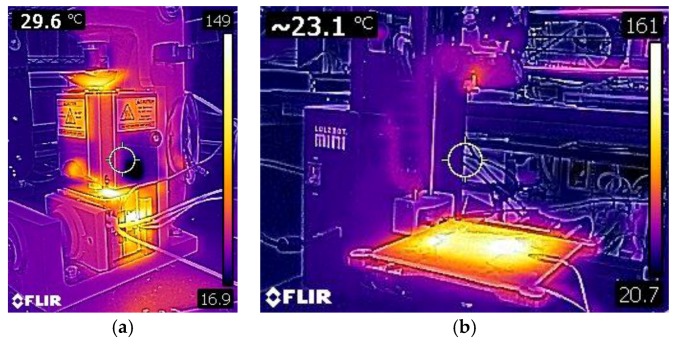
Injection molding setup heating to the set temperature (150 °C) (**a**) and a modified 3D printer (Lulzbot Mini) to print precursor Aquivion filament heating to set temperatures (the extruder is set to 260 °C and the bed is set to 180 °C) (**b**).

**Figure 5:**
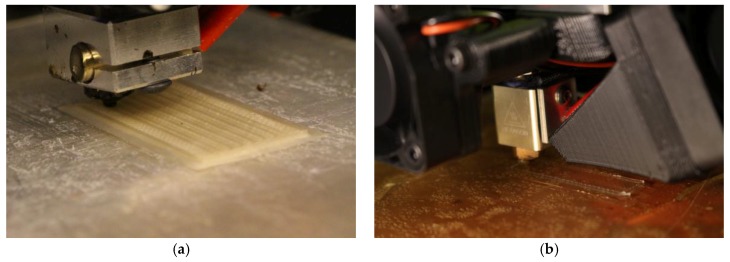
A precursor Nafion membrane printed with inner channels for potential implementation of liquid sensors (**a**) and a precursor Aquivion membrane being printed in typical dimensions for IPMC applications (**b**).

The un-activated form of the ionomers is available as precursor pellets. These has been used in extrusion [25] and hot-pressing [26]. More recently, newer fabrication methods have been developed: injection molding [27] and 3D printing [11,28]. For all of these methods, it is important to understand how these ionomers behave with thermal loads. Although the precursor pellets can melt, if not heated properly or thoroughly during the process, degradation can occur. For the injection molding process, the mold that the pellets enter needs to be at a similar temperature as the chamber where they are heated. This is done by heating the mold to the same temperature (Figure 4a). Finding the correct temperature to set these two environments will help optimize the process. For 3D printing, the bed temperature needs to be high enough to encourage adhesion, while also allowing the print to cool down to have good layering (Figure 4b and Figure 5). The best temperatures for Aquivion are 260 °C for the extruder and 180 °C for the bed [11]. The best temperatures for Nafion are 290 °C for the extruder and 180 °C for the bed [28]. 

To date, there has been no thorough study of the thermal characteristics of Nafion and Aquivion precursor pellets. For researchers, the key points that need to be clarified are the thermal degradation temperature, viscosity, melting temperature, and glass transition. The objective of this study is to provide a clear guide for the ionomer precursor pellets in order to optimize various thermal-based fabrication processes. This can be done by conducting various tests to characterize the precursor ionomers.

## 2. Methods

For these tests, Nafion precursor pellets with an equivalent weight (EW) of 970 g/eq (C.G. Processing, Chadds Ford, PA, USA) and Aquivion precursor pellets with an EW of 870 g/eq (P87S-SO_2_F, Solvay, Bolatte, Italy) were used. To characterize Nafion and Aquivion precursor pellets, a series of tests were conducted. First, the chemical structure of the two were studied using a Fourier Transform-Infrared Spectroscopy (FT-IR, IRTracer-100, Shimadzu, Kyoto, Japan). The thermal degradation temperature was measured using a Thermogravimetric Analyzer (TGA, Q500, TA Instruments, New Castle, DE, USA) in a nitrogen atmosphere. The heat rate was 10 °C/min and temperature range was set to start at room temperature up to 650 °C. The balance and sample purge flow of N_2_ gas were set to 40 mL/min and 60 mL/min, respectively. Rheological tests were conducted with a rotational rheometer (MCR 300, Anton-Paar, Graz, Austria) using a parallel-plate geometry (D = 25 mm) to measure the change in shear viscosity as temperature increases and the melting temperature. From the steady shear test, the shear stress was measured as a function of shear rate in the range of 0.01–10 (1/s), while in an oscillatory shear measurement, an angular frequency from 0.6 to 600 (rad/s) was applied under a constant strain of 1%. To measure the glass transition range, samples were tested in a Dynamic Mechanical Analyzer (DMA, Pyris Diamond, Perkin Elmer, Waltham, MA, USA) that has the ability to control the temperature of the test environment. The samples were annealed in the oven overnight at 140 °C, following the procedure described by Tao [29]. The heat rate was set to 1 °C/min and the heat range was set to start at 20 °C to 100 °C. The tests were conducted at a frequency of 1 Hz with an applied force of 50 mN.

## 3. Results

**Figure 6:**
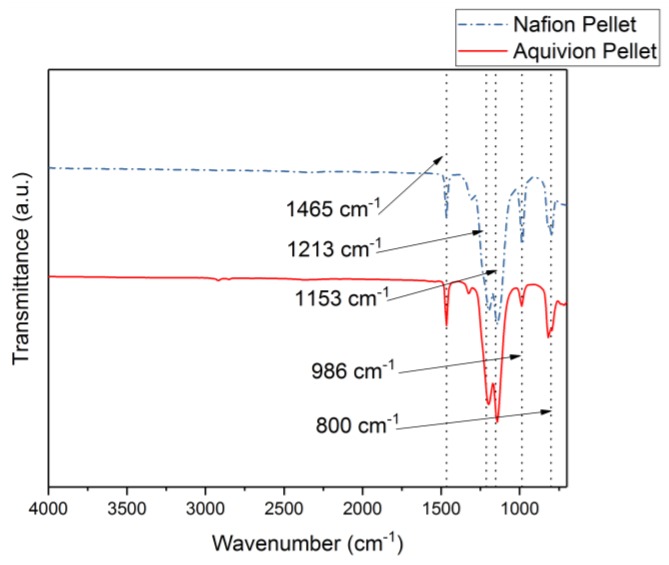
FT-IR results comparing Nafion and Aquivion precursor pellets.

**Figure 7:**
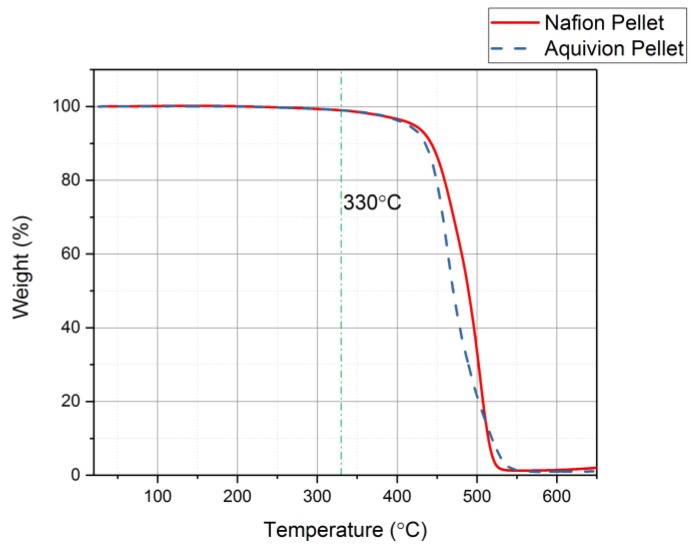
Thermogravimetric Analyzer (TGA) results showing that both precursor ionomers have the same thermal degradation temperature at 330 °C.

**Figure 8:**
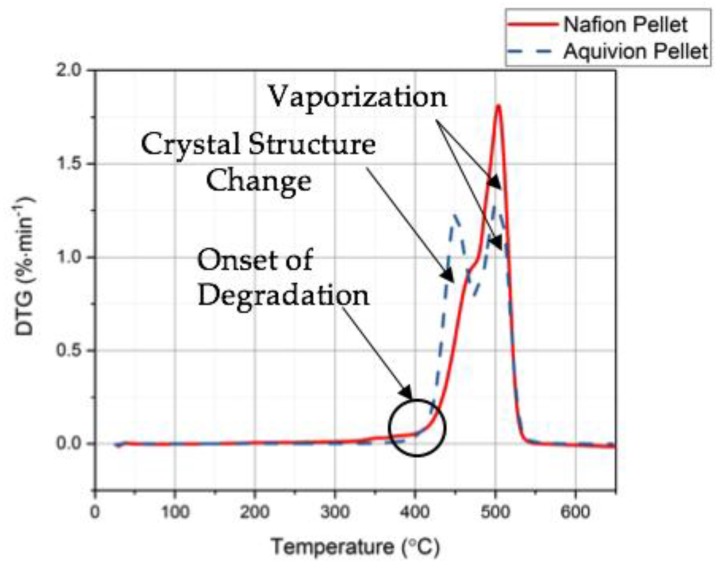
Differential Thermogravimetric (DTG) results from the TGA tests for precursor Nafion and precursor Aquivion.

First, the chemical structures were analyzed with the FT-IR. Figure 6 shows the transmittance of the two precursor ionomers, which have similar structures based on this plot. The peaks at 1465 cm^−1^, 1213 cm^−1^, 1153 cm^−1^, 986 cm^−1^, and 800 cm^−1^ correlate to C–F, symmetric CF_2_, asymmetric CF_2_, C–F_3_, and S–O bonds, respectively [11]. The thermal degradation temperature of the two ionomers is around 330 °C (Figure 7). Both degrade at almost the same rate. To further investigate the degradation of the two precursor ionomers, the Differential Thermogravimetric (DTG) curves have been plotted (Figure 8). It can be seen that degradation more accurately begins at around 400 °C. The peak for precursor Nafion is most likely due to vaporization. The peaks for precursor Aquivion are most likely correlated to a change in crystal structure, followed by vaporization.

**Figure 9:**
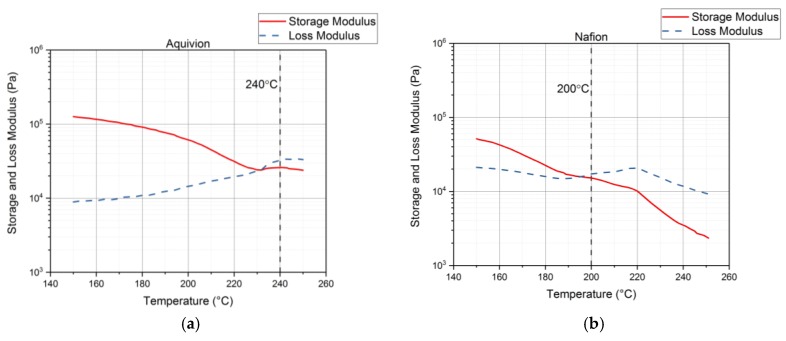
Results from the rheology tests showing the melting temperature of precursor Aquivion (**a**) and precursor Nafion (**b**).

**Figure 10:**
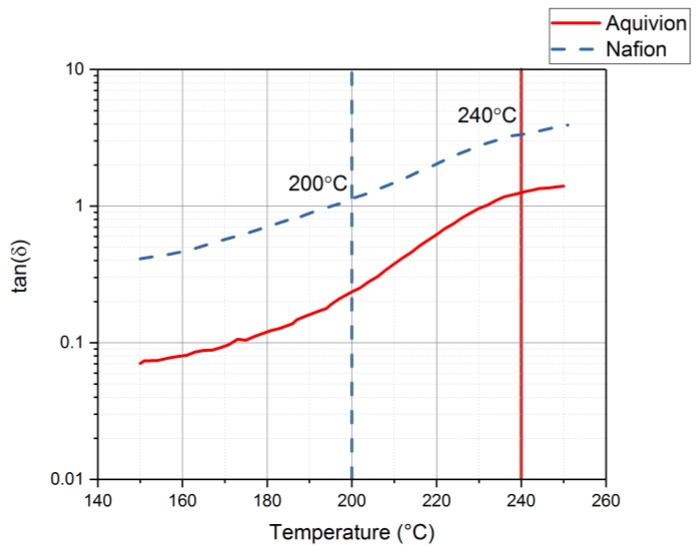
Results for tan(δ) from the rheology tests to understand the viscous behavior of precursor Aquivion and precursor Nafion.

**Figure 11:**
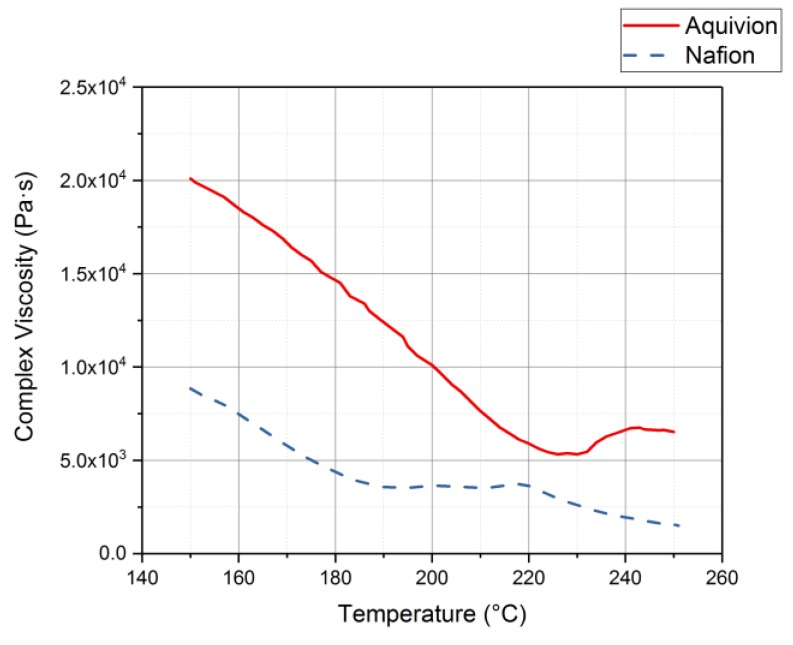
Complex viscosity results from the rheology tests conducted on the precursor-ionomers.

**Figure 12:**
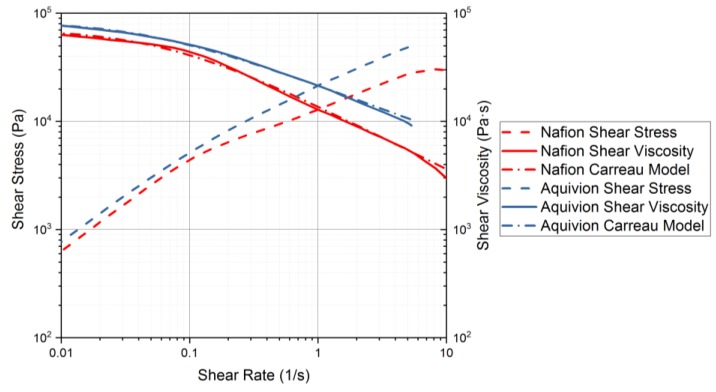
Rheology tests with applied shear rate to measure the shear stress and shear viscosity of the precursor-ionomers. Precursor Nafion was tested at 200 °C and precursor Aquivion was tested at 240 °C.

**Figure 13:**
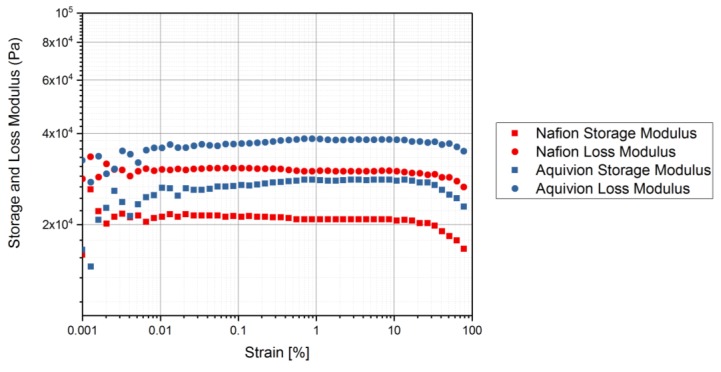
Rheology tests with an applied strain to measure the storage and loss modulus of the precursor-ionomers. Precursor Nafion was tested at 200 °C and precursor Aquivion was tested at 240 °C at a frequency of 1 Hz.

**Figure 14:**
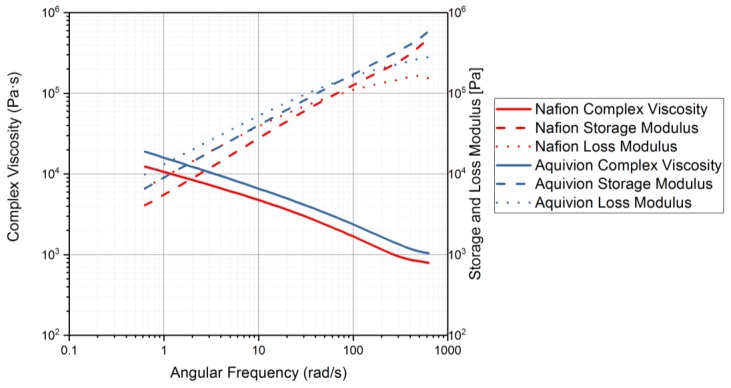
Rheology tests with an applied angular frequency to measure the complex viscosity and storage and loss modulus. Precursor Nafion was tested at 200 °C and precursor Aquivion was tested at 240 °C.

Rheology tests were run to gather data about the ionomers under different conditions. The data for the storage modulus and loss modulus for each ionomer and where the curves cross, denotes where the polymer begins to melt. From Figure 9, it can be seen that precursor Aquivion’s melting temperature is about 240 °C and for precursor Nafion, the melting temperature is about 200 °C. The melting temperature for precursor Aquivion matches that provided by Solvay [24]. The plots in Figure 9 show that the two ionomers exhibit partially crystalline behavior, because after the two lines intersect, the loss modulus is larger than the storage modulus [30]. To further analyze the results, the ratio between storage and loss modulus is tan(δ) and this provides information about the viscous behavior for the two precursor samples. When tan(δ) is less than 1, the sample has more of a gel-like behavior and when it is greater than 1, the behavior is more liquid-like [31]. In Figure 10, it can be seen that the values for precursor Nafion are greater than 1 at about 200 °C and the values for precursor Aquivion are greater than 1 at about 240 °C, which matches well with what is seen in Figure 9. It was also of interest to observe the change in viscosity as the temperature increases. Notably, precursor Aquivion has a higher viscosity throughout the temperature sweep compared to precursor Nafion (Figure 11). With an applied shear rate (precursor Nafion was tested at 200 °C and precursor Aquivion was tested at 240 °C at a frequency of 1 Hz), it can be seen that precursor Aquivion has higher shear stress and shear viscosity than precursor Nafion (Figure 12). Both exhibit similar trends as the shear rate increases. With an applied strain (precursor Nafion was tested at 200 °C and precursor Aquivion was tested at 240 °C at a frequency of 1 Hz), precursor Aquivion still has higher storage and loss moduli (Figure 13). Based on the results, it can be noted that the two exhibit liquid-like characteristics, because the loss modulus is higher than the storage modulus [30]. As the angular frequency increases (precursor Nafion was tested at 200 °C and precursor Aquivion was tested at 240 °C), again precursor Aquivion has higher complex viscosity and storage and loss moduli than precursor Nafion (Figure 14). The angular frequencies between 1 and 1000 rad/s simulates an extrusion process and above ~100 rad/s simulates an injection process [30]. The material characteristics for ionomers in 3D printing and injection molding process can be seen in Figure 14.

**Figure 15:**
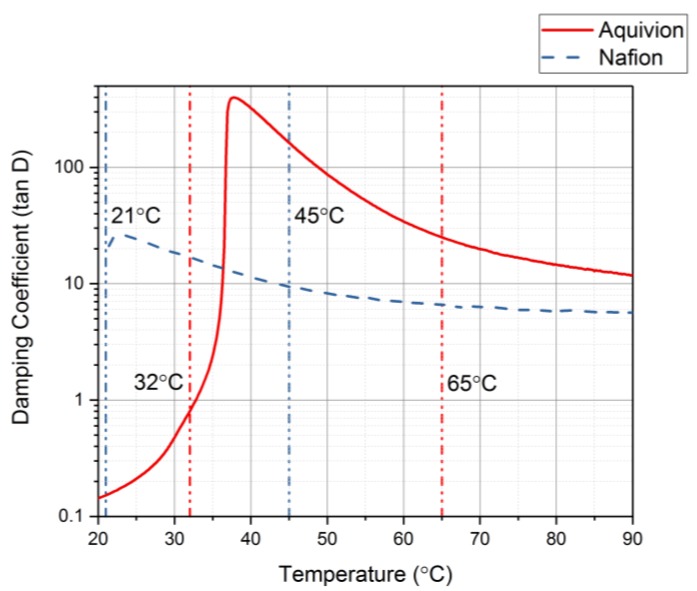
Damping coefficient as the temperature increases to find the glass transition temperature range for the precursor ionomers.

**Figure 16:**
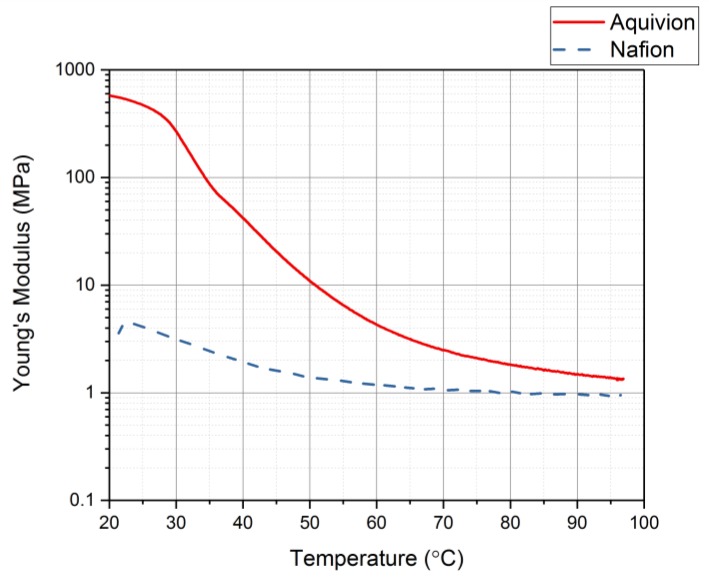
Young’s Modulus as the temperature increases for each precursor ionomer.

The DMA was used with thermal control to identify the glass transition range for each precursor ionomer [29]. This can be done by plotting the damping coefficient values as the temperature increases. Figure 15 shows the damping coefficient for precursor Aquivion and precursor Nafion. Precursor Aquivion has a larger range than precursor Nafion (32–65 °C and 21–45 °C, respectively). From this plot, it can also be noted that the precursor Aquivion sample is significantly stiffer than precursor Nafion. To verify this, the Young’s Modulus for each was studied (Figure 16). It is clear from this plot that the precursor Aquivion sample is much stiffer at room temperature than precursor Nafion, but decreases to the same value as it is heated. To show how different the two types of ionomers are, Table 1 presents a comparison of the damping coefficients and Young’s Moduli of the activated and precursor forms. The activated forms are generally stiff, but it is interesting to note the vast difference in the precursor forms. Precursor Aquivion is significantly stiffer than the activated form, while precursor Nafion is significantly softer than the activated form. These differences further verify that precursor Aquivion may be easier to work with since there is a larger range in which the polymer becomes rubbery to liquid.

## 4. Discussion 

Both of the precursor ionomers exhibit partially crystalline behavior based on the results shown in Figure 9. Using Figure 14, it is possible to see how the material will behave under certain angular frequencies that simulate different processes. During the applied angular frequency test, the loss modulus was higher than the storage modulus for both precursor ionomers, indicating liquid-like behavior at the chosen correlating temperatures (Figure 14). 

On the other hand, according to the shear viscosity as a function of precursor Nafion and precursor Aquivion (Figure 8), both ionomers show a non-Newtonian behavior (shear thinning) where the shear viscosity decreases with shear rate. Note that the shear thinning property implies solid-like behavior at a high shear rate with potential chain alignment under a shear. The flow behavior of the two precursor materials can be fitted using a weighted non-linear regression to a modified-Carreau model [32].

(1) $$\eta =\frac{\eta_{0}}{{\left[1+\left(\lambda \dot{\gamma }\right)\right]}^{\left(1-n\right)}}$$

where η refers to the shear rate dependent shear viscosity, $\eta_{0}$ is the zero-shear viscosity, *λ* is the characteristic time, $\dot{\gamma }$ is a shear rate, and *n* is a dimensionless parameter. The slope of the power-law region with shear-thinning behavior is governed by (1 − *n*). The different flow behaviors with different degrees of shear-thinning can be considered with various values of *n* in Equation (1). The value of *n* will be 1 for Newtonian fluids while typical shear thinning behavior is shown when *n* < 1. The modified-Carreau model was well fitted with the experimental data as shown in Figure 10 with the calculated values of $\eta_{0}$, λ, and *n* as illustrated in Table 2. The values for *n* for the two ionomers show that precursor Nafion has more shear-thinning abilities than precursor Aquivion, since it has a lower *n* value. From the shear stress measurement under different strains (Figure 14), both precursor ionomers illustrate viscoelastic characteristics within the strain range of 1 and 10%, which is used to conduct in oscillatory measurement. In addition, the complex viscosity obtained from the dynamic oscillation test shows a similar trend to the shear viscosity of the two precursors where the shear viscosity of precursor Aquivion is higher than that of precursor Nafion. 

The precursor Nafion samples were very soft at room temperature. This is verified by the data from the DMA, showing that the glass transition temperature of precursor Nafion starts at room temperature. The melting temperature is also lower than expected (200 °C). This data verifies what is noted by Grot in his discussion about fluorinated ionomers where he mentions that films made from Nafion precursor pellets are soft at room temperature and stretch easily [18]. This is verified by the data gathered from the DMA. The precursor Nafion sample is very soft at room temperature.

Precursor Aquivion on the other hand has a higher glass transition and melting temperature. It seems that precursor Aquivion is a more thermally stable polymer. This can make it easier to work with since it is more moldable. It was noted throughout different processes, such as hot-pressing and filament extrusion, that precursor Aquivion was easier to use and mold into the desired shape. 

## 5. Conclusions

A thorough study of the behaviors of precursors Nafion and Aquivion was undertaken. The melting temperature of both ionomers was established. The ionomers have similar thermal degradation temperatures. The viscosity of the precursor ionomers was studied versus temperature, strain rate, and angular frequency. Precursor Aquivion has higher viscosity than precursor Nafion. The shear viscosity was fitted to a modified-Carreau model and showed that the precursor ionomers both have shear-thinning abilities. Precursor Nafion has more shear-thinning abilities due to its lower *n* value than precursor Aquivion. Precursor Nafion is much softer at room temperature, which was verified using the DMA to find that its glass transition range, which starts at room temperature and with a Young’s Modulus at around 3 MPa. Precursor Aquivion is significantly stiffer at room temperature (~574 MPa) and a larger glass transition range. 

The gathered data showed that precursor Aquivion may be better to use in processes such as extrusion, hot pressing, and 3D printing. This is because precursor Aquivion is more able to be molded into shape after being heated. Precursor Nafion becomes much more liquid like, making it difficult to shape, such as in filament extrusion. However, the study provides sufficient information and evidence for researchers to use either precursor ionomer in a thermal-based fabrication process.

## Figures and Tables

**Table 1:** Comparison of the mechanical characteristics of activated and precursor forms of the ionomers at room temperature.

Mechanical Characteristics	Aquivion		Nafion	
	Activated	Precursor	Activated	Precursor
Damping Coefficient (tan D)	0.122 ± 0.000085	0.144 ± 0.00061	0.058 ± 0.00012	21.1 ± 8.74
Young’s Modulus (MPa)	293 ± 0.14	574 ± 2.82	329 ± 0.42	3.57 ± 2.588

**Table 2:** Parameter variables of modified-Carreau model (Equation (1)) for two precursor ionomers.

Ionomer	*η*_0_ (Pa s)	λ (s)	*n*	r^2^
Nafion	7.1 × 10^4^	15.4	0.41	0.945
Aquivion	8.4 × 10^4^	21.5	0.56	0.977
